# Take the NEXT STEP: Practical Tools and Strategies for Clinical Trials Work in Nursing Homes

**DOI:** 10.1093/geroni/igaf122.450

**Published:** 2025-12-31

**Authors:** Kathleen Unroe, Debra Saliba, Jerry Gurwitz

## Abstract

Nursing home residents have been historically excluded from clinical trials. Numerous challenges have been described to conducting research in nursing homes, including barriers to recruitment, methodological difficulties, and a need for pilot funding preparatory to larger trials. The National Institute on Aging funded the $15.6 million NEXT STEPs (Nursing home EXplanatory clinical Trials: Supporting Transformation by Enhancing Partnerships) U24 initiative to directly address these challenges. The goal of NEXT STEPs is to expand the number and quality of multi-site clinical trials in the nursing home setting. The perspectives and participation of interdisciplinary clinical providers and researchers are critical to inform the launch and activities of this innovative network. This symposium will highlight several of the activities and resources developed to date by the NEXT STEPs Network, with a goal of providing practical tools and guidance for researchers and partners in nursing home research.

